# Trajectories of Depressive Symptoms and Neighborhood Changes from Adolescence to Adulthood: Latent Class Growth Analysis and Multilevel Growth Curve Models

**DOI:** 10.3390/ijerph17061829

**Published:** 2020-03-12

**Authors:** Hyunjung Lee, Lorena M. Estrada-Martínez

**Affiliations:** 1 Oak Ridge Institute for Science and Education (ORISE), Oak Ridge, TN 37831, USA; hyunjung.lee0001@gmail.com; 2 School for the Environment, University of Massachusetts Boston, Boston, MA 02125, USA

**Keywords:** neighborhood changes, racial/ethnic composition, depression, neighborhood socioeconomic status, health disparities, latent class growth analysis, multilevel growth curve models

## Abstract

The role of neighborhood socioeconomic status (SES) and racial/ethnic composition on depression has received considerable attention in the United States. This study examines associations between trajectory patterns of neighborhood changes and depressive symptoms using data from Waves I-IV of the National Longitudinal Study of Adolescent to Adult Health. We used latent class growth analysis to determine the number and distribution of person-centered trajectories for neighborhood characteristics, and multilevel growth curve models to examine how belonging to each class impacted depression trajectories from ages 13 to 32 among non-Hispanic Whites (NHW), non-Hispanic Blacks (NHB), Hispanics, and non-Hispanic Others (NHO). The distribution of neighborhood SES classes across racial/ethnic groups suggests significant levels of economic inequality, but had no effect on depressive symptoms. A more complex picture emerged on the number and distribution of racial/ethnic composition latent class trajectories. Compared to NHB peers who lived in predominantly NHW neighborhoods from adolescence to adulthood, NHBs in more diverse neighborhoods had lower risk for depressive symptoms. Conversely, Hispanics living in neighborhoods with fewer NHWs had higher risk for depressive symptoms. Among NHOs, living in neighborhoods with a critical mass of other NHOs had a protective effect against depressive symptoms.

## 1. Introduction

There is an extensive body of research on how neighborhoods can minimize and exasperate depression among individuals [1,2,3]. The impact of neighborhood socioeconomic status (SES) and racial/ethnic composition on the mental health of people of color in the United States (USA) has received significant attention, with mixed findings [1,2,3,4,5,6,7,8,9]. Neighborhood environments have been identified as important sources of both stress and protection for individuals [10,11]. Although many studies have examined the associations between these environments and depression, there is limited information on long-term depression trajectories among people who move to different types of neighborhoods from childhood through adulthood.

Researchers have used life course frameworks to study depression trajectories across life stages. Allen and colleagues argue that depression at early ages suggests stressors that may continue to affect individuals’ health into adulthood [10]. In fact, in the USA the prevalence of depressive symptoms among youth is significant. In 2017, 30% of non-Hispanic White (NHW), 29% of non-Hispanic Black (NHB), and 34% of Hispanic youth reported sadness and hopelessness in the previous two weeks [12]. Evidence suggests there is an age curve where adolescents and young adults in all racial and ethnic groups experience higher rates of depression than older adults. However, approximately 17.7 million adults also reported experiencing at least one major depressive episode (MDE) in 2017 [13]. Nearly 8% of NHW adults had a MDE, while 5.4% of NHB and Hispanics, and 4.4% of Asians were diagnosed with depression [14]. The influence of individuals’ race/ethnicity on social pathways throughout life cannot be overstated. Race/ethnicity are primary determinants of where a person lives, goes to school, and works throughout their life course. People in different racial/ethnic groups start out and continue to move through vastly different environments over the course of their lives [15,16,17]. Despite these racial/ethnic- and age-related trends, few researchers have examined how people of different racial/ethnic groups experience changes in the racial/ethnic composition of their neighborhood environments over the life course, and how in turn these influence long-term depression trajectories.

### 1.1. Changes in Neighborhood Socioeconomic Status and Depression across Race/Ethnicity

Scholarly research on the relationship between depression and neighborhood SES has produced varied results. In meta-reviews, Mair et al. and Richardson et al. highlighted that most studies have found low SES, most commonly measured as economic disadvantage or deprivation, to be associated with higher depressive symptoms (or conversely neighborhood affluence and wealth indictors to be associated with lower risks) [1,3]. More recently however, Fang et al. and Goldstein et al. found no association between neighborhood SES and depression, and Brazil and Clark, and Estrada-Martínez et al. found associations among some groups, but not others [4,5,6,7]. Researchers have argued that neighborhood SES can affect depression through several mechanisms, including social connections with neighbors, availability and quality of health care (especially mental health) and recreational facilities, quality of housing, educational and occupational opportunities, and feelings of (un)safety that impose stress on the lives of their residents [1,2,18]. In low SES neighborhoods, these stressors may add pressure on already strained protective social connections or social supports, which in turn increases the risk for depression [1,2,18].

Few studies have examined the effect of changes in neighborhood SES over time on long-term depressive symptoms. Using data from the Moving to Opportunity (MTO) study, Graif et al. found that mental health improved for females who moved to low poverty neighborhoods compared to those who did not move or those who moved to a neighborhood of similar SES [19]. It also found that among those who moved to low poverty neighborhoods, both the immediate and surrounding neighborhood-level poverty were key in improving mental health outcomes [19]. Fong et al. found that there was no association between changes in neighborhood SES and depressive symptoms among Australian adolescents, adults, or older adults [6]. Brazil and Clark examined the association between depressive symptoms and changes in neighborhood poverty from adolescence to emerging adulthood [7]. Using Waves I (ages 11–21), III (ages 18–26), and IV (ages 24–32) of the National Longitudinal Study of Adolescent to Adult Health (Add Health), they examined whether participants who moved to more affluent areas (moved up), to higher poverty areas (moved down), or did not change also experienced changes in their risks for depression. They found that moving into more affluent neighborhoods from Waves I to III was associated with fewer depressive symptoms among males, while moving to more affluent neighborhoods from Waves I to IV reduced depressive symptoms among females. These results suggest that experiencing improvements in neighborhood SES from adolescence to adulthood has an impact on depression. However, their analytic approach did not include a fuller account of the spectrum of changes in neighborhood contexts. Brazil and Clark calculated three different estimates for changes from Waves I to III, Waves I to IV, and Waves III to IV, rather than creating a single curve that explored the full changes comprising Waves I to IV [7]. Moreover, their analysis did not consider how changes in neighborhood racial/ethnic composition may impact youth’s future mental health.

### 1.2. Changes in Neighborhood Racial/Ethnic Composition and Depression across Race/Ethnicity

Residential segregation is a fundamental cause of racial disparities in health [17,20]. Williams and Collins have argued that residential segregation leads to health disparities by reinforcing economic inequality, thus creating unfavorable environments for health [17]. For example, low SES in racially segregated communities of color ultimately means that residents have limited access to educational and occupational opportunities, health care facilities, are more likely to live in poor quality housing and are exposed to higher rates of crime and violence [17]. Although the above characteristics are detrimental to ones’ mental health, researchers have also found evidence that strong social support networks in highly segregated neighborhoods can improve the mental health outcomes of its residents [1,2,3,4,8,21]. Thus, the effect of neighborhood SES on depression may depend on both the neighborhoods’ racial/ethnic composition and the residents’ race/ethnicity. Given that people of color disproportionately reside in lower SES, segregated neighborhoods and the conflicting evidence on the effect of residential segregation on NHW vis-à-vis people of color, it is important to understand how neighborhood contexts shape mental health disparities by race/ethnicity [20,21,22,23,24].

Some studies have examined the association between neighborhood racial/ethnic composition and depressive symptoms by individuals’ race/ethnicity [1,4,8,9,25,26,27,28] with mixed results. Notably, Mair et al. described two hypotheses explaining the relationship between neighborhood racial/ethnic composition and depression: (1) the *ethnic density hypothesis* and (2) the *residential segregation hypothesis* [23]. The ethnic density hypothesis [29] proposed that, due to social support and social cohesion, persons residing in neighborhoods with a higher density of their own race/ethnicity have better mental health outcomes compared to persons residing in those with lower density. The residential segregation hypothesis [24,30,31] explained that due to structural racism, higher concentrations of certain racial/ethnic groups is associated with worse mental health. Mair et al. argue that the ethnic density hypothesis applies to immigrant groups, such as Hispanics and Asians, while the residential segregation hypothesis applies to African Americans [23].

Similarly, Portes and Zhou have argued that immigrant groups experience three possible economic and residential mobility trajectories [32]. The first path is increasing integration into the white middle class (the dominant, majority group); the second path is assimilation into the urban, (presumably black) underclass, which leads to downward social and economic mobility; and the third path is “selective assimilation,” where the group engages in deliberate preservation of their culture and values, and in upward economic integration in the form of ethnic enclaves. As a result, some groups have experienced downward mobility with increases in poverty, residential segregation, and negative health outcomes in subsequent generations [33], while others experienced upward economic mobility while living in highly segregated neighborhoods [34]. Building upon these, we expect unique patterns by race/ethnicity to stem from differences in the social environments experienced. At the heart of these frameworks is the paradox that, for people of color, living in minority neighborhoods translates into experiencing less interpersonal racism, but living in a racially and economically segregated neighborhood also means experiencing more structural racism, and hence, more material deprivation.

Prior studies have found that different neighborhood elements have unique effects for specific racial/ethnic groups [1,4,8,9,23,26,27]. For NHW, some studies found an association between the proportion of NHW residents in the neighborhood and depression [25,27], while others did not [5,23]. For NHB, Mair et al. found that living in a neighborhood with a higher proportion of Black residents was associated with higher depressive symptoms [23], while English et al. and Wight et al. found that NHB residents in primarily NHW neighborhoods had higher risk for depressive symptoms than those in more integrated neighborhoods [25,28]. For Hispanics and Asians, Mair et al. found that living in a neighborhood with a higher proportion of the same race/ethnicity was associated with fewer depressive symptoms [23]. Similarly, Ostir et al. and Shell et al. found that among Mexican Americans, increases in the Mexican American population in their neighborhoods was associated with a decrease in depressive symptoms [8,35]. Studies suggest that what matters about neighborhoods is different for different racial/ethnic groups. Noting the vast increases in neighborhood SES that accompany living in predominantly NHW neighborhoods as compared with minority neighborhoods [36], the impact of being a racial minority in a majority NHW neighborhood and in neighborhoods with higher SES may shift over time [37].

### 1.3. The Current Study

The present study builds and expands upon the work of Brazil and Clark to examine a broader array of changes in neighborhood SES, including upward, downward, and no economic mobility. While Brazil and Clark looked at changes in neighborhood SES from adolescence to adulthood, they primarily focused on gender differences and the role of gendered life transitions on the risk for depression [7]. Our study focuses on the potential differences in depressive symptoms by race/ethnicity. We also extend the work of Mair et al. [23] by examining changes in neighborhood racial/ethnic composition, among people of different racial/ethnic background. Using a person-centered approach [38], we determine the multiple longitudinal trajectory patterns of neighborhood changes with regard to SES and racial/ethnic composition from adolescence into adulthood. We use latent class growth analysis (LCGA) to describe the neighborhood change trajectories among a racially diverse, nationally representative sample of youth from the mid-1990s through 2008. LGCA produces a more comprehensive representation of changes people experience with regard to their neighborhoods. We then used multilevel growth curve models (MGCM) to examine the association between trajectory patterns of neighborhood changes and trajectory patterns of depressive symptoms from age 13 to 32.

## 2. Methods and Measures

The study population consists of 9421 participants of all four waves of the In-Home interviews of the National Longitudinal Study of Adolescent to Adult Health (Add Health) [39]. Participants were recruited from 132 middle and high schools that were representative of schools in the country. Student selection was stratified by grade and sex, and weighted to represent adolescents in the USA enrolled between 7th and 12th grade during the 1994–95 academic year. At Wave I, participants were 11–21 years old, 13–22 at Wave II, 18–27 at Wave III, and 24–35 at Wave IV. Because the number of participants aged 11–12 and 33–35 was small, we limited analyses to participants aged 13 to 32. Add Health developed multilevel, longitudinal weights, applicable only to respondents who participated in all four waves, which we used in our analysis to mitigate bias due to differential attrition. Table 1 presents a detailed description of the sample and the distribution of the outcome and key independent variables by race/ethnicity.

**Depression Symptoms**. Depressive symptoms were measured at baseline and at each subsequent wave with a 9-item version (Cronbach’s α ≥ 0.79) of the Center for Epidemiological Studies Depression Scale (CES-D) [40]. Sample items include *You felt sad* and *You were bothered by things that usually don’t bother you*. Responses referred to the prior week and ranged from 0 = *Never or rarely* to 3 = *Most or all of the time.* Summed scores ranged from 0–27, with higher scores meaning more depressive symptoms; scores >5 indicated elevated depressive symptoms [41]. in order to ensure confidentiality of sensitive questions during the in-home interviews, participants responded to these questions using an Audio Computer Assisted Self-Interview (ACASI).

**Race/Ethnicity.** Respondents self-identified their Hispanic ethnicity and racial identity in separate questions. We used this information to create mutually exclusive categories of non-Hispanic Whites (NHW), non-Hispanic Blacks (NHB), Hispanics of any race (Hispanics), and non-Hispanic others (NHO).

**Neighborhood Characteristics.** We based neighborhood indicators on U.S. Census data corresponding to participants’ home addresses at each wave, with neighborhoods defined as census tracts. We developed a standardized measure of neighborhood SES that included indicators of income (proportion of families with incomes >$75,000), employment and occupation (proportion of individuals in managerial or professional occupations, unemployment rate [reversed]), education (proportion of adults with college degrees), poverty (proportion of families living in poverty [reversed]), and at-risk populations (proportion of youth 16–19 years of age not in school, labor force, or military, and has not completed high school/GED [reversed]) [42,43]. Higher scores indicate a more favorable neighborhood SES (Cronbach’s α > 0.84). We also assessed the neighborhood race/ethnicity with four continuous variables indicating the proportion of non-Hispanic Whites (%NHW), non-Hispanic Blacks (%NHB), Hispanics (%Hispanics), and non-Hispanic others (%NHO). These continuous variables were used to conduct the LCGA; classes from these analyses were then used as predictor variables in the MGCM (details are discussed under Statistical Analysis). 

**Individual and Family Demographic Variables.** Interviewers noted participants’ gender as male (referent group [Ref]) or female at Wave I. We calculated age by subtracting each wave’s interview date from the participants’ birthdate. We also included indicators for household composition and household income [44], at Wave I. Because SES during adulthood is related to adults’ mental health, including depression, we also included a standardized measure of household SES at Wave IV based on participants’ job rank, highest educational attainment, and poverty ratio [45].

### 2.1. Statistical Analyses

To answer our research questions, we proceeded in two primary analytic steps: (1) LCGA to determine the number of and describe the person-centered trajectory classes for each neighborhood characteristic, and (2) MGCM in which we used the classes identified in Step 1 as predictors of depressive symptoms among different racial/ethnic groups. Given the intersecting roles of race/ethnicity and socioeconomic status at both the individual and neighborhood levels, we stratified our analyses by the respondents’ race/ethnicity. Thus, we identified trajectory patterns of neighborhood change for each racial/ethnic group (NHW, NHB, Hispanics, NHO), and then examined how these were associated with trajectory patterns of depressive symptoms among each group in individual analyses.

#### 2.1.1. Step 1. Latent Class Growth Analysis

We used LCGAs to identify patterns of trajectories in respondents’ neighborhood (a) SES, (b) %NHW, (c) %NHB, (d) %Hispanics, and (e) %NHO from Waves I through IV. LCGA captures unobserved heterogeneity over time and allows us to identify a different latent trajectory for each class to produce categorical latent variables that more closely reflect lived experiences, as well as the mean of growth parameters for each subgroup [46,47]. We used the Type = MIXTURE syntax to use mixture model algorithms in MPlus 8.3 [48]. In the MODEL syntax, we assigned the same model and free estimates across classes by using the “%overall%” syntax. Then, we specified the appropriate factor loadings, which are time-varying outcome variables that correspond to the time intervals for the intercept and slope. The zero time score for the slope growth factor at Wave I (1994–1995) defines the intercept growth factor as an initial status factor: one for Wave II in 1996, eight for Wave III in 2001, and 13 for Wave IV in 2008. Although the time period between Waves I and II is too short to show feasible changes in neighborhood characteristics, we decided to include Wave II for consistency of the study periods between the LCGA and the MGCM. We fixed the within-class variances to zero, which is an unconditional latent class model without covariates that allows for clearer identification of classes and less computational burden [46]. We specified the number of random sets of starting values as 1000, the number of final optimizations as 100, and the initial round of optimizations as 20 to ensure successful convergence. We started with a three-class model and proceeded to increase the number of classes until we found the best fit model according to the Akaike Information Criterion (AIC), Bayesian Information Criterion (BIC), adjusted BIC, the entropy value, and the Lo, Mendell, and Rubin Likelihood Ratio Test (LMR-LRT). We also considered sample size, theoretical justification, and interpretability to decide the number of classes.

#### 2.1.2. Step 2. Multilevel Growth Curve Model

We used MGCM to examine the association between trajectory patterns of depression symptoms and trajectory patterns of neighborhood changes, as identified through the LCGA in Step 1. We assessed participants’ baseline levels (i.e., intercepts) and changes over time (i.e., slopes) in depressive symptoms from adolescence to adulthood. Age was centered at 16, the Wave I mean, to provide a meaningful interpretation of the intercept. As an extension of multilevel models [49], multiple observations of an individual were nested within a person, who in turn was nested within a school. Schools were the primary sampling unit in Add Health. MGCM analyses were conducted in Stata 15 MP [50], which allowed us to incorporate multilevel, longitudinal weights at the individual and school levels developed by Add Health to mitigate bias due to differential attrition [51]. We used the user-written program PWIGLS Method 2 to create group-specific multilevel scaled weights [52]. We assigned near-zero values at the individual level of the scaled multilevel weights to respondents who were not part of the model’s focus (e.g., non-NHWs in the model for NHWs) [51]. We fit an unconditional model to calculate the intra-class correlation coefficient (ICC), which indicates the variance attributed to participants’ growth trajectories (Level 1 within-individual variation) and the differences between participants’ trajectories (Level 2 between-individual variation) [49]. Results indicate that over 38.2% of the variance in depressive symptoms can be attributed to within-individual differences, while 1.5% can be attributed to between-individual differences.

To assess individual baseline levels and changes in trajectories, and to examine patterns of influence on those trajectories, we used 3-level random effects models, where Level 1 represents individual change; Level 2 is a vector of fixed individual and household variables, the latent neighborhood classes, and random effects for linear age; and Level 3 accounts for the school-level clustering. To maximize our sample, we addressed missing items in individual-level covariates at Wave I by using multiple imputation with chained equations (MICE) for panel data [53] to generate 10 imputations. Most missing data were on household income at Wave I (21.5%) and adult SES at Wave IV (19.8%). Missing values on other variables ranged from 0% to 1.5%. The final MGCM results are based on pooled estimates from separate growth curves in each of the imputed data sets [54]. Records from less than 150 participants contained missing data on neighborhood characteristics; hence, we used non-imputed data in Step 1 of the analysis.

## 3. Results

As shown in Table 1, NHWs had lower depressive symptoms than any other racial/ethnic group. Groups also differed in income, household composition, and neighborhood characteristics. NHWs and NHOs had higher income and neighborhood SES than NHBs or Hispanics. NHWs (70.6%) had the highest proportion of two-parent households, while NHBs had the highest proportion of single parent-households (30.6%) Unsurprisingly, at Wave I, NHWs resided in neighborhoods with the highest level of %NHW (89.1%) than any group. NHBs resided in neighborhoods with the highest %NHB (53.4%) and a high proportion of %NHW (41.2%); Hispanics’ neighborhoods had the highest %Hispanics (32.7%), and NHOs had the highest neighborhood %NHO (17.6%).

### 3.1. Latent Class Growth Analysis

Using the model fit criteria discussed above, we first chose the best class solutions among each racial/ethnic group. Then, we categorized the latent class of each variable based on the number of classes and their percentile distribution at Wave I. Baseline neighborhood race/ethnicity percentiles >0.8 were considered Very High, >0.6 to 0.8 were High, >0.4 to 0.6 were Medium, >0.25 to 0.40 Medium-Low, >0.05 to 0.25 Low, and ≤0.05 were Very Low. Neighborhood SES was represented as: >1.7 Very High, >0.9 to 1.7 High, >0.1 to 0.9 Med, > −0.5 to 0.1 Med-Low, >−1.3 to -0.5 Low, and <−1.5 to −1.3 Very Low. We also characterized the direction of the trajectory as Decrease, No Change, and Increase based on whether the averages changed from the original percentile category to another, as well as the direction of any changes. In light of the subsequent MGCM analysis and for ease of interpretation, we rearranged the classes to follow their overall categories as described above. There were differences in the number of classes, their distributions, and changes over time across race/ethnicity.

**Figure 1:**
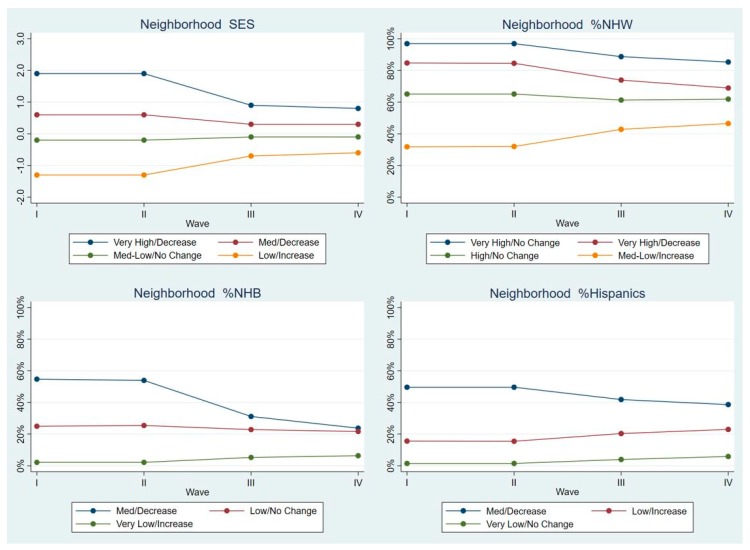
Latent class means for each neighborhood characteristic by Wave, Non-Hispanic Whites.

Non-Hispanic Whites. As seen in Figure 1, there were four LGCA classes for neighborhood SES: Very High/Decrease (6.8% of NHW were in this class), Med/Decrease (28.6%), Med-Low/No Change (49.2%), Low/Increase (15.5%). There were also four classes of %NHW: Very High/No Change (65.2%), Very High/Decrease (21.9%), High/No change (10.3%), Med-Low/Increase (2.6%). This mirrored their distribution in minority neighborhoods. For %NHB and %Hispanics there were three classes. For %NHB, Med/Decrease (2.5%), Low/No Change (8.1%), and Very Low/Increase (89.4%); for %Hispanics these were Med/Decrease (0.9%), Low/Increase (10.2%), Very Low/No Change (88.9%).

**Figure 2:**
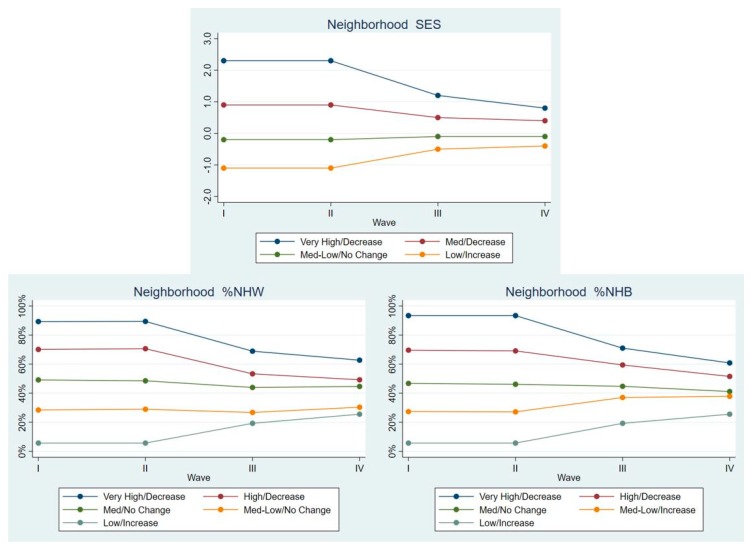
Latent class means for each neighborhood characteristic by Wave, non-Hispanic Blacks.

Non-Hispanic Blacks. As seen in Figure 2, there were four LCGA classes for neighborhood SES, and five classes for %NHW and %NHB in the neighborhoods. Neighborhood SES classes reflected Very High/Decrease (2.9% of NHB was in this class), Med/Decrease (16.7%), Med-Low/No Change (57.9%), and Low/Increase (22.6%) neighborhoods. LCGA classes on the %NHW in the neighborhood were Very High/Decrease (15.4%), High/Decrease (17.2%), Med/No Change (16.3%), Med-Low/No change (19.2%), and Low/Increase (31.9%). The LCGA classes on the %NHB in the neighborhood were Very High/Decrease (29.4%), High/Decrease (19.3%), Med/No Change (13.2%), Med-Low/Increase (19.7%), Low/Increase (18.5%).

**Figure 3:**
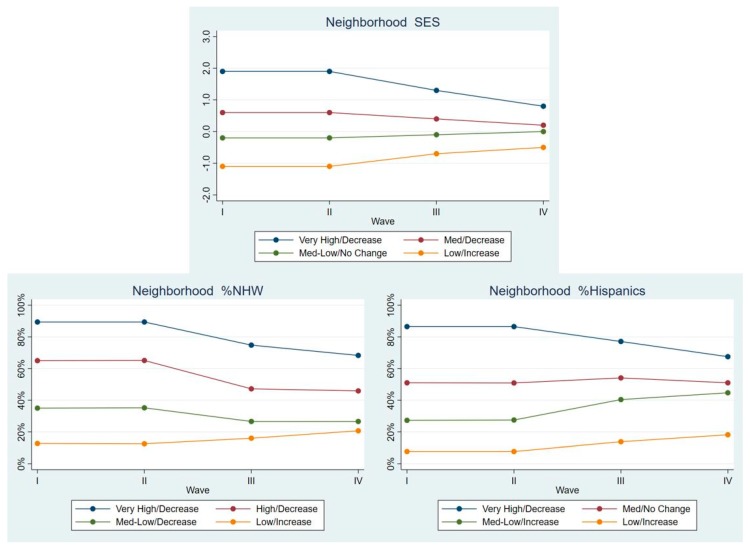
Latent class means for each neighborhood characteristic by Wave, Hispanics.

Hispanics. As seen in Figure 3, there were four LCGA classes for neighborhood SES, and the %NHW and %Hispanics in the neighborhoods. For neighborhood SES, they were Very High/Decrease (8.0% of Hispanics were in this class), Med/Decrease (29.5%), Med-Low/No Change (34.4%), and Low/Increase (28.1%). For %NHW, they were Very High/Decrease (27.9%), High/Decrease (23.5%), Med-Low/Decrease (24.3%), and Low/Increase (24.4%). For %Hispanics, they were Very High/Decrease (16.0%), Med/No Change (17.7%), Med-Low/Increase (24.8%), and Low/Increase (41.5%).

**Figure 4:**
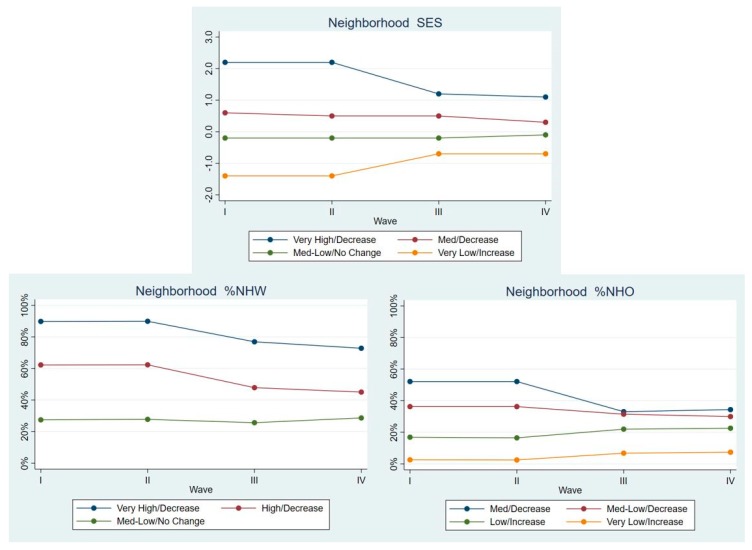
Latent class means for each neighborhood characteristic by Wave, non-Hispanic Others.

Non-Hispanic Others. As seen in Figure 4, there were four LCGA classes for neighborhood SES, and %NHO in the neighborhood. For neighborhood SES, they were Very High/Decrease (6.2% of NHO was in this class), Med/Decrease (25.7%), Med-Low/No Change (14.5%), and Very Low/Increase (53.6%). For %NHW, there were three classes: Very High/Decrease (37.6%), High/Decrease (26.8%), and Med-Low/No Change (35.6%). For %NHO, there were four classes: Med/Decrease (10.5%), Med-Low/Decrease (17.6%), Low/Increase (20.8%), and Very Low/Increase (51.1%).

### 3.2. Multi-Level Growth Curve Model

#### Results from the MGCM are presented in Table 2

Non-Hispanic Whites. Depressive symptoms followed a quadratic trajectory. Mirroring the full sample, scores decreased in their early 20s and increased substantially by their late 20s. Males had lower scores at age 16 than females. We did not find statistically significant effects of neighborhood SES class trajectories among this group. However, both higher %NHW (β = −3.26, 95% CI: −6.21, −0.32) and %NHB (β = −2.75, 95% CI: −5.25, −0.25) in the respondents’ neighborhoods were associated with lower depressive symptoms at baseline. No other neighborhood class trajectory was significant among this group.

Non-Hispanic Blacks. Depressive symptoms followed a linear decrease, and males had lower scores at baseline than their female counterparts. We found a significant effect of the %NHW latent trajectory classes. Compared to NHB respondents in the “Very High/Decrease” latent trajectory class —that is, those who lived in neighborhoods where over 80% of the population was NHW during their adolescence, and then moved to neighborhoods with significantly lower proportions—NHB respondents in the “High/Decrease” (β = −1.20, 95% CI: −2.24, −0.16) and “Med-Low/No Change” (β = −2.53, 95% CI: −4.90, −0.15) %NHWs had lower risks for depressive symptoms. No other neighborhood class trajectory was significant among this group.

Hispanics. Depressive symptoms followed a linear decrease, and males had lower scores at baseline than their female counterparts. Higher %NHW at Wave I were also associated with higher depressive symptoms at baseline (β = 5.65, 95% CI: 1.92, 9.39). Compared to respondents in the “Very High/Decrease” %NHW latent trajectory class (reference group), Hispanic respondents in the “Med-Low/Decrease” %NHW (β = 2.26, 95% CI: 0.21, 4.30) and the “Low/Increase” %NHW (β = 3.14, 95% CI: 0.41, 5.87) latent trajectory classes had significantly higher risks for depressive symptoms. No other neighborhood trajectory class was significant among this group.

Non-Hispanic Others. Depressive symptoms followed a linear decrease and there were no gender differences at baseline. Higher %NHOs at Wave I were also associated with higher depression symptoms at baseline (β = 7.22, 95% CI: 2.72, 11.72). NHOs are the only group who were affected by the latent trajectory for their own proportion in neighborhood. Compared to respondents in the “Med/Decrease” latent class (reference group), those in “Med-Low/Decrease” (β = 2.50, 95% CI: 0.31, 4.69) and “Very Low/Increase” classes (β = 4.77, 95% CI: 1.58, 7.97) had higher risks for depressive symptoms. No other neighborhood class trajectory was significant among this group.

## 4. Discussion

### 4.1. Changes in Neighborhood Socio-Economic Environments and Depression Trajectories

NHW, NHB, Hispanic, and NHO respondents fell into four latent classes of neighborhood SES trajectories. The large variations in average neighborhood SES, as well as in its changes, suggests substantive economic inequalities across respondents’ race/ethnicity. However, these changes did not translate into associations between neighborhood socio-economic characteristics and depressive symptoms among any racial/ethnic group. The lack of association is consistent with previous studies [5,6,7]. Several factors may partially explain this lack of association, including a waning of influence as people age, or its relative importance to more proximate socioeconomic indicators, such as income, occupation or level of education [2,3,55]. However, in light significant neighborhood SES inequality by respondents’ race/ethnicity, this non-significant finding highlights the importance of racial/ethnic composition as it relates to long-term mental health outcomes.

### 4.2. Changes in Neighborhood Racial/Ethnic Composition and Depression Trajectories

This study’s findings suggest an important, but complex effect of the changes in the racial/ethnic composition of ones’ neighborhood on depressive symptoms. Results among Hispanics are consistent with the racial segregation hypothesis, while those among NHO are consistent with the ethnic density hypothesis. Interestingly, it was not the %NHB that was most relevant for NHB respondents (which is the traditional emphasis of the racial segregation hypothesis), but rather the concentration of %NHW at different points in their life. It is likely that NHB respondents who resided in integrated neighborhood from adolescence into adulthood (i.e., “Med-Low/No Change” %NHW class) were exposed to more diversity of experiences, but had enough people like them to ease the process of “othering” and the psychological tensions (e.g., harassment/violence, micro-aggressions) that come with it. Similarly, NHB respondents who started out in predominantly %NHW neighborhoods and moved to more integrated neighborhoods may have experienced an ease of the same psychological tensions.

NHW respondents overwhelmingly lived in hyper-segregated neighborhoods as adolescents, and some gradually moved into slightly less segregated neighborhoods as they grew older. NHW respondents lived among other NHW people and few people of other groups, even as they grew older. For example, respondents in the “Very High/No Change” class started out in neighborhoods that were nearly 100% NHW and ended up in neighborhoods that were about 85% NHW. Similarly, those in the “Very High/Decrease” class crossed the categorical thresholds but lived in neighborhoods that were about 70% NHW by adulthood. The trajectories of %NHW was also parallel to those of %NHB and %HSP. Almost 90% of NHW respondents lived in neighborhoods where less than 5% of the population was NHB or Hispanic, with only a 3% increase by the time they reached adulthood.

Non-Hispanic Whites. Consistent with Mair et al. [23], we did not find an association between neighborhood racial/ethnic composition changes and depressive symptoms among NHWs. However, the significance of the baseline proportions of NHWs and NHBs in the neighborhood (both protective) may have different implications about these extremes [24,25,30]. The findings of a negative association between depressive symptoms and the %NHB at baseline among NHWs is surprising since the findings are opposite with racial segregation theory, which argues that higher segregation of NHB leads to poor health status. The findings might indicate positive social interactions in integrated neighborhoods. Wickrama, Noh and Bryant found a non-significant trend that an increase in the proportion of non-Whites in the neighborhood decreases depressive symptoms [56]. However, others have found that White youth in racially diverse neighborhoods can experience social alienation, powerlessness, fear, and anxiety [57]. Our finding that Whites have a decrease in depressive symptoms as neighborhood NHB increases at Wave I requires further investigation.

Non-Hispanic Blacks. NHB respondents lived in more racially diverse neighborhoods than their NHW counterparts. Nearly 50% of NHB respondents started out in the “Very High” or “High” %NHB neighborhood classes, and there was a clear trend towards living in more integrated neighborhoods as they got older. Results among this group do not support the racial segregation nor the ethnic density hypothesis. We found that NHBs who lived in predominantly NHW neighborhoods as adolescents and moved into neighborhoods with fewer whites as adults had lower risk for depressive symptoms than those who stayed in neighborhoods with high levels of %NHW. Similarly, respondents in the “Medium-Low/No Change” class of %NHW in the neighborhoods were at lower risk for depressive symptoms. However, we found no evidence that the %NHB in the neighborhoods was a determinant of depression trajectories. These findings are consistent with previous studies [23,25,27,28], that have found no relationships between concentration of NHB and CES-D scores. Results in the “Med-Low/No Change” category suggest that living in relatively integrated neighborhoods throughout adolescence and young adulthood may be protective for the mental health of Black youth. Among Black people who grew up in predominantly White neighborhoods, moving into more integrated neighborhoods can be protective and reduce risk of depressive symptoms compared to those who continued to live in hyper-segregated White neighborhoods. This might be explained by previous findings that the percent of Whites in a neighborhood had an indirect effect on depressive symptoms through racial discrimination [24,28]. Accordingly, Black people in White-dominant neighborhoods might experience more racial discrimination, resulting in increased depressive symptoms [28].

Hispanics. The majority of Hispanic respondents experienced a reduction in the proportion of NHWs in their neighborhoods as they entered adulthood, while 66.3% of their neighborhoods experienced an increase in the proportion of Hispanics. Although %NHW at Wave I was associated with higher risk for depressive symptoms during adolescence, Hispanic respondents in the “Med-Low/Decrease” or “Low/Increase” %NHW classes were also more likely to experience depressive symptoms than those in the “Very High/Decrease” %NHW class. This finding is consistent with the racial segregation hypothesis and could be reflective of segmented assimilation, where some groups become increasingly economically and spatially isolated from NHWs. For example, Hispanics that live in neighborhoods with high economic and racial segregation can also experience hypervigilance from local law enforcement, which can indirectly create more chronic stressors that result in depressive. Awareness of, and actions against, this type of structural racism are especially relevant at this time of heightened anti-immigrant federal policies.

Non-Hispanic Others. NHO respondents, during their adolescence, seem to have been more integrated into higher %NHW neighborhoods, since 63.5% resided in “Very High” or “High” %NHW classes. As they got older, non-Hispanic others were more likely to move into neighborhoods with higher %NHO. However, growing up in relatively integrated neighborhoods but moving into neighborhoods with fewer NHOs as they got older was associated with higher risks of depressive, as was growing up in neighborhoods with low numbers of NHOs to begin with. Our findings on NHOs were consistent with previous a study using the ethnic density hypothesis for Asians [23].

## 5. Conclusions

There are several limitations in this study. First, there is limited information on the number of moves, and places of residence, of all participants between waves. Although Add Health provides geocoded for neighborhood characteristics at the time of the interviews, it is not possible to directly assess all potential changes in types of neighborhoods that can occur during the entire 13-year period. Moreover, this study is focused on understanding the exposure of respondents, and not changes in the neighborhoods per se. Thus, it is possible that a respondent could have remained in the same neighborhood for the entire study period, but that neighborhood experienced rapid sociodemographic transitions, effectively changing the respondent from one analytic category to another. More research is needed to differentiate the neighborhood effect from geographical relocation, compared to the change in the same neighborhood over time. Second, we defined census tracts as neighborhoods, given their availability in Add Health and consistency with other studies of neighborhood contexts and mental health However, this administrative definition of boundaries does not capture the residential environment or border area, or residents’ perceptions of their neighborhoods’ boundaries. Third, we measured depressive symptoms using a 9-item version (Cronbach’s α ≥ 0.79) of CES-D, which includes reliable and valid criteria in terms of predictability and the correlation between self-reported depressive symptoms with clinical ratings of severity of depression [40]. However, since CES-D is based on a survey rather than a clinical diagnosis, it might be sensitive to the questions’ wording and could be affected by interviewers; this is especially relevant for adolescents responding to sensitive questions [7,40]. Fourth, the threshold naming latent trajectory classes of neighborhood for SES and racial/ethnic composition is arbitrary, even though it is similar with the quintile base. While we found robustness in latent trajectory classes depending on the changes in threshold, further study may investigate different thresholds to characterize each latent class.

Despite these limitations, this study contributes to our understanding of the complexity of how changes in both racial/ethnic composition and neighborhood SES relate to depressive symptoms in diverse racial-ethnic groups as they emerge into adulthood. Our study used a novel research design, particularly the integration of LCGA and MGCM to contribute to determine the number and distribution of person-centered trajectories for each neighborhood characteristic, and to examine how belonging to each class impacted depressive symptoms. We demonstrated that living in more integrated neighborhoods was protective for Black respondents vis-à-vis living in higher SES neighborhoods where they were more likely to be a more visible minority, while living in segregated neighborhoods was associated with higher depressive symptoms for Hispanics, but lower depressive symptoms for NHO. Conventional theory dichotomously applied residential segregation hypothesis to NHW and NHB and ethnic density hypothesis to Hispanics and NHO, but results from this study show that it is not simply ethnic density or residential segregation, but a combination of both depending on where one falls in America’s racial/ethnic power hierarchy. In future research, we recommend replication of the analysis for Hispanic or non-Hispanic Asian subgroups, given their heterogeneity of national origins and culture. Moreover, it is important to study on experiences of discrimination as the mechanisms that link racial/ethnic composition and neighborhood SES to depressive symptoms and other mental health outcomes. Our study highlights the importance of considering race/ethnicity at multiple levels when developing policies that aim to reduce poverty area concentration. Health care providers must consider the particular contexts and needs of youth of color as they transition into adulthood in light of in light of their social positions within their neighborhoods, the kinds of strains and opportunities these provide, and how these may be impacting peoples’ mental health.

## Figures and Tables

**Table 1:** Descriptive statistics and tests of difference by racial/ethnic groups.

Variable (Mean/%, SE)	NHW	NHB	Hispanics	NHO	Test
Sample size (%)	5316 (56.43)	1967 (20.88)	1445 (15.34)	693 (7.36)	
Depressive symptoms					
Wave I	5.40 (0.11)	6.48 (0.18)	6.39 (0.20)	6.61 (0.33)	R^2^ = 0.01, F (3, 126) = 14.27 ***
Wave II	5.33 (0.09)	6.35 (0.16)	6.75 (0.21)	6.41 (0.29)	R^2^ = 0.02, F (3, 126) = 22.18 ***
Wave III	4.28 (0.07)	5.10 (0.18)	5.17 (0.17)	5.24 (0.27)	R^2^ = 0.01, F (3, 126) = 14.36 ***
Wave IV	4.93 (0.07)	6.05 (0.2)	5.58 (0.17)	5.46 (0.25)	R^2^ = 0.01, F (3, 126) = 13.43 ***
Age					
Wave I	15.46 (0.13)	15.70 (0.19)	15.60 (0.22)	15.65 (0.23)	
Wave II	16.38 (0.13)	16.66 (0.19)	16.54 (0.21)	16.60 (0.24)	
Wave III	21.81 (0.13)	22.07 (0.19)	22.06 (0.21)	22.03 (0.24)	
Wave IV	28.31 (0.13)	28.60 (0.19)	28.55 (0.21)	28.6 (0.23)	
Male (%)	49.80 (0.01)	48.75 (0.02)	50.96 (0.02)	54.95 (0.03)	χ^2^ (3) = 5.82
Income (in thousands)	49.89 (1.86)	29.13 (1.72)	34.29 (2.07)	43.98 (3.20)	R^2^ = 0.05, F (3, 126) = 28.25 ***
Adult SES	0.02 (0.02)	0.11 (0.04)	0.03 (0.04)	-0.02 (0.06)	R^2^ = 0.001, F (3, 126) = 1.95
Household composition (%)					
2 parents	70.61 (0.01)	33.47 (0.02)	54.75 (0.02)	56.30 (0.04)	χ^2^ (12) = 1131.61 ***
2 parents & adult kin	9.39 (0.01)	10.45 (0.01)	18.63 (0.02)	23.02 (0.04)	
1 parent	14.73 (0.01)	30.61 (0.02)	14.64 (0.02)	9.27 (0.02)	
1 parent & adult kin	3.34 (0.00)	16.55 (0.01)	9.49 (0.01)	5.94 (0.02)	
Adult kin, no parents	1.93 (0.00)	8.93 (0.01)	2.50 (0.01)	5.47 (0.01)	
Neighborhood SES at WI	0.14 (0.08)	-0.55 (0.08)	-0.18 (0.09)	0.15 (0.1)	R^2^ = 0.09, F (3, 126) = 28.64 ***
%NHW at WI	89.10 (0.01)	41.23 (0.03)	52.07 (0.04)	60.29 (0.05)	R^2^ = 0.47, F (3, 126) = 81.53 ***
%NHB at WI	5.54 (0.01)	53.35 (0.03)	8.69 (0.01)	9.3 (0.02)	R^2^ = 0.51, F (3, 126) = 64.64 ***
%Hispanics at WI	3.31 (0.00)	3.56 (0.01)	32.72 (0.05)	12.84 (0.02)	R^2^ = 0.38, F (3, 126) = 18.36 ***
%NHO at WI	2.02 (0.00)	1.68 (0.00)	6.55 (0.01)	17.63 (0.05)	R^2^ = 0.21, F (3, 126) = 5.80 **

Note: SE=Standard Error, NHW = non-Hispanic Whites, NHB = non-Hispanic Blacks, NHO = non-Hispanic others, WI = Wave I. Group differences were tested by ANOVA and Pearson’s chi-squared test, ** *p*< 0.01, *** *p* < 0.001.

**Table 2:** Results of multi-level growth curve models of depressive symptoms (Betas and 95% Confidence Intervals).

Variable	NHW	NHB	Hispanics	NHO
Intercept	**12.6 (9.09, 16.11)**	**11.17 (6.26, 16.08)**	2.78 (−3.16, 8.73)	1.35 (−6.80, 9.49)
Age	**−0.34 (−0.48, −0.19)**	**−0.06 (−0.09, −0.02)**	**−0.10 (−0.13, −0.07)**	**−0.10 (−0.13, −0.07)**
Age^2^	**0.01 (0.00, 0.01)**			
Male	**−1.21 (−1.41, −1.01)**	**−0.94 (−1.42, −0.46)**	**−1.01 (−1.40, −0.63)**	−0.40 (−1.16, 0.36)
Income	**−0.43 (−0.64, −0.21)**	**−0.39 (−0.65, −0.14)**	−0.18 (−0.71, 0.35)	−0.17 (−0.75, 0.42)
Adult SES	**0.14 (0.00, 0.29)**	0.14 (−0.12, 0.40)	−0.04 (−0.33, 0.25)	0.17 (−0.26, 0.60)
Household Composition				
2 parents	Ref.	Ref.	Ref.	Ref.
2 parents & adult kin	**0.58 (0.14, 1.01)**	0.32 (−0.29, 0.93)	0.22 (−0.42, 0.86)	0.46 (−0.28, 1.21)
1 parent	0.19 (−0.29, 0.66)	**0.60 (0.04, 1.15)**	0.76 (−0.93, 2.46)	0.52 (−0.33, 1.37)
1 parent & adult kin	0.66 (−0.05, 1.38)	**0.67 (0.07, 1.28)**	0.49 (−0.81, 1.79)	**1.75 (0.31, 3.18)**
Adult kin, no parents	0.91 (−0.14, 1.96)	**0.87 (0.07, 1.67)**	0.76 (−1.09, 2.61)	**2.07 (0.58, 3.57)**
SES at Wave I	0.21 (−0.16, 0.58)	0.37 (−0.37, 1.10)	0.33 (−0.36, 1.02)	0.51 (−0.15, 1.18)
SES latent classes				
Very High/Decrease	Ref.	Ref.	Ref.	Ref.
Med/Decrease	0.03 (−0.48, 0.54)	0.87 (−1.19, 2.93)	0.04 (−1.47, 1.55)	0.02 (−0.92, 0.95)
Med-Low/No change	0.24 (−0.44, 0.93)	1.34 (−1.32, 4.00)	0.04 (−1.68, 1.75)	−0.24 (−1.71, 1.23)
Med-Low/Increase				
Low/Increase	0.87 (−0.15, 1.90)	2.17 (−0.92, 5.26)	0.95 (−1.32, 3.22)	
Very Low/Increase				0.22 (−1.00, 1.43)
NHW at Wave I	**−3.26 (−6.21, −0.32)**	−2.02 (−5.94, 1.90)	**5.65 (1.92, 9.39)**	0.99 (−4.04, 6.02)
NHW latent classes				
Very High/No Change	Ref.			
Very High/Decrease	−0.21 (−0.66, 0.25)	Ref.	Ref.	Ref.
High/No Change	−0.37 (−1.23, 0.49)			
High/Decrease		**−1.20 (−2.24, −0.16)**	1.22 (−0.23, 2.67)	1.98 (−0.35, 4.30)
Med/No Change		−0.99 (−2.67, 0.68)		
Med-Low/Decrease			**2.26 (0.21, 4.30)**	
Med-Low/Increase	−1.00 (−2.67, 0.67)			
Med-Low/No change		**−2.53 (−4.90, −0.15)**		3.20 (−0.45, 6.84)
Low/Increase		−2.47 (−5.48, 0.54)	**3.14 (0.41, 5.87)**	
NHB at Wave I	**−2.75 (−5.25, −0.25)**	−1.57 (−4.74, 1.61)		
NHB latent classes				
Very High/Decrease		Ref.		
High/Decrease		−0.07 (−0.91, 0.78)		
Med/Decrease	Ref.			
Med/No Change		−1.27 (−2.67, 0.13)		
Med-Low/Increase		−1.53 (−4.25, 1.19)		
Low/No Change	0.26 (−0.42, 0.95)			
Low/Increase		−0.98 (−3.1, 1.13)		
Very Low/Increase	0.39 (−0.32, 1.10)			
HSP at Wave I	−2.38 (−7.09, 2.33)		2.44 (−2.13, 7.00)	
HSP latent classes				
Very High/Decrease			Ref.	
Med/Decrease	Ref.			
Med/No Change			0.68 (−1.04, 2.41)	
Med-Low/Increase			0.59 (−1.77, 2.95)	
Low/Increase	1.02 (−0.21, 2.26)		0.63 (−2.09, 3.35)	
Very Low/No Change	1.05 (−0.53, 2.63)			
NHO at Wave I				**7.22 (2.72, 11.72)**
NHO latent classes				
Med/Decrease				Ref.
Med-Low/Decrease				**2.50 (0.31, 4.69)**
Low/Increase				2.99 (−0.33, 6.30)
Very Low/Increase				**4.77 (1.58, 7.97)**

Note: NHW = non-Hispanic Whites, NHB = non-Hispanic Blacks, NHO = non-Hispanic others. **Boldface = *p* < 0.05.**
